# Differential Adsorption of Ochratoxin A and Anthocyanins by Inactivated Yeasts and Yeast Cell Walls during Simulation of Wine Aging

**DOI:** 10.3390/toxins7104350

**Published:** 2015-10-26

**Authors:** Leonardo Petruzzi, Antonietta Baiano, Antonio De Gianni, Milena Sinigaglia, Maria Rosaria Corbo, Antonio Bevilacqua

**Affiliations:** Department of the Science of Agriculture, Food and Environment (SAFE), University of Foggia, Via Napoli 25, Foggia 71122, Italy; E-Mails: leonardo.petruzzi@unifg.it (L.P.); antonietta.baiano@unifg.it (A.B.); antonio.degianni@unifg.it (A.D.G.); milena.sinigaglia@unifg.it (M.S.); mariarosaria.corbo@unifg.it (M.R.C.)

**Keywords:** ochratoxin A, anthocyanins, yeasts, adsorption, cell wall, wine aging

## Abstract

The adsorption of ochratoxin A (OTA) by yeasts is a promising approach for the decontamination of musts and wines, but some potential competitive or interactive phenomena between mycotoxin, yeast cells, and anthocyanins might modify the intensity of the phenomenon. The aim of this study was to examine OTA adsorption by two strains of *Saccharomyces cerevisiae* (the wild strain W13, and the commercial isolate BM45), previously inactivated by heat, and a yeast cell wall preparation. Experiments were conducted using Nero di Troia red wine contaminated with 2 μg/L OTA and supplemented with yeast biomass (20 g/L). The samples were analyzed periodically to assess mycotoxin concentration, chromatic characteristics, and total anthocyanins over 84 days of aging. Yeast cell walls revealed the highest OTA-adsorption in comparison to thermally-inactivated cells (50% *vs*. 43% toxin reduction), whilst no significant differences were found for the amount of adsorbed anthocyanins in OTA-contaminated and control wines. OTA and anthocyanins adsorption were not competitive phenomena. Unfortunately, the addition of yeast cells to wine could cause color loss; therefore, yeast selection should also focus on this trait to select the best strain.

## 1. Introduction

The adsorption of wine components by yeasts, yeast lees, and inactivated yeast fractions is of increasing interest in winemaking, for managing fermentations, wine stabilization, and aging processes [1,2]. Several studies have reported on the interaction of yeast cells with a variety of wine compounds, including anthocyanins [3,4,5,6] and flavan 3-ol derivatives [2,7], aromatic substances [1,8], sulfur products [9] or undesirable components, such as octanoic and decanoic acids [10], 4-ethylphenol [11,12,13], geosmin [14], and some pesticides commonly used in vineyards [15].

Currently, there is an increasing interest towards yeast adsorption/removal of ochratoxin A (OTA) [16]. OTA is a mycotoxin produced as a secondary metabolite by several toxigenic molds belonging to *Aspergillus* and *Penicillum* species. It possesses nephrotoxic, immunosuppressive, teratogenic, and carcinogenic (group 2B) properties [17]. Since the vintage of 2006, with the adoption of Regulation CE 123/05, the level of OTA in commercial wines cannot exceed 2 μg/L, but many trade agreements usually require lower limits (e.g., 0.5 μg/L) [18].

Several researchers studied the removal of OTA by yeasts during alcoholic fermentation [19,20,21,22,23,24,25,26,27]. Several winemaking practices involve a prolonged contact between yeasts or yeast-derived products and wine, thus suggesting that yeast cells could play a significant role in OTA removal also at the end of the fermentation process [28]. Núñez *et al.* [29] and Petruzzi *et al.* [28] studied the effect of aging on the removal of OTA in a model wine system by *Saccharomyces cerevisiae* and a commercial yeast-cell wall preparation. However, in real wine OTA adsorption might be modified by other molecules that could be also adsorbed by yeast cell wall. Therefore, a topic of great concern relies upon the potential interactions between OTA, yeasts, and anthocyanins, being these last compounds largely responsible for the color of red wines [30]. At present, only some speculations are available in the literature. For example, García-Moruno *et al.* [31] and Caridi *et al.* [19] suggested the existence of competition phenomena between wine polyphenols and OTA for the same binding sites on the surface of the yeast cells. Cecchini *et al.* [20] suggested that yeast mannoproteins, known to react with polyphenols, could interact with OTA, too. Thus, the aim of this paper was to investigate the ability of inactivated *S. cerevisiae* strains and a commercial yeast-cell wall preparation to reduce OTA content in Nero di Troia red wine over 84 days of aging. In addition, potential competitive or interlinking phenomena between OTA, yeast cells, and anthocyanins were evaluated through the analysis of anthocyanic compounds adsorbed on yeast cells in wines added with OTA.

## 2. Results

### 2.1. OTA-Removal by Inactivated Yeasts and Yeast Cell Walls

**Figure 1:**
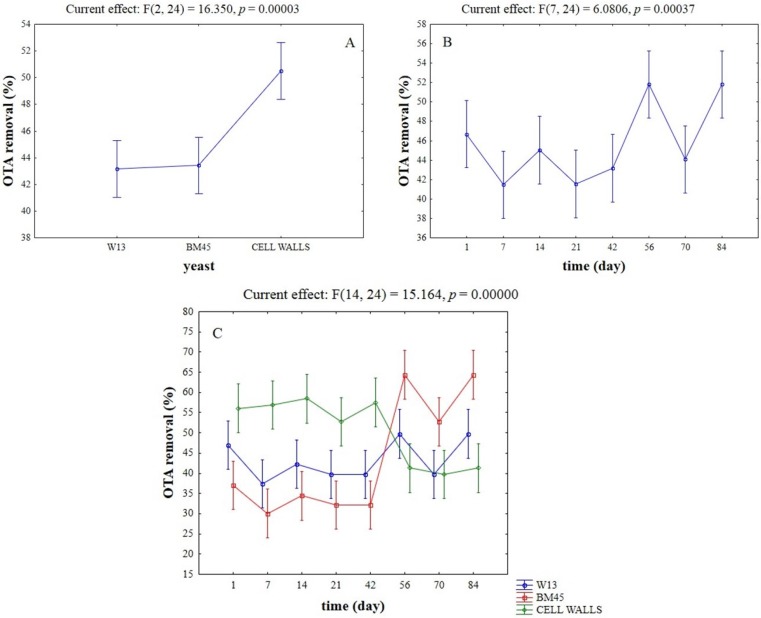
Effective hypothesis decomposition of two-way ANOVA for the effects of yeast/time on OTA removal (%). (**A**) Effect of yeasts/yeast cell walls; (**B**) effect of time; and (**C**) interaction. Vertical bars denote 95% confidence. For each effect on the upper side of figure there are the *F*-test and the relative degrees of freedom.

The concentrations of OTA in wines with or without treatments with yeasts or yeast cell walls are shown in Table 1; the initial content of toxin was 2 μg/L. The coefficient of variation was from 0 to 4.51%. These results were modeled as percentages of removed OTA and used as input values for a two-way ANOVA (Figure 1). The analysis of variance shows the significant influence of the kind of adsorbent (yeasts or yeast cell walls), time, and their interactive effect (*p* < 0.01). Hypothesis decomposition (Figure 1A) pinpointed that the highest reduction was found for the yeast cell wall preparation (*ca*. 50% of initial concentration), whereas the results for the strains W13 and BM45 were not significantly different (OTA removed by 43%). Concerning the effect of time, the highest level of reduction (*ca*. 48%–55%) was found after 56 and 84 days (Figure 1B). Finally, a strong interactive effect was recovered for the strain BM45 (increase of OTA removal over time) and for yeast cell walls (decrease of OTA removal over time), whereas the performance of the strain W13 did not experience significant changes (Figure 1C).

**Table 1:** Concentrations of OTA (µg/L) in control wines and samples supplemented with yeasts or yeast cell walls. Mean values ± standard deviation.

Time (Day)	Control	W13	BM45	Cell Walls
1	1.98 ± 0.00	1.35 ± 0.03	1.45 ± 0.06	1.27 ± 0.03
7	1.96 ± 0.03	1.43 ± 0.03	1.51 ± 0.03	1.25 ± 0.05
14	1.92 ± 0.03	1.35 ± 0.04	1.43 ± 0.03	1.21 ± 0.05
21	1.94 ± 0.00	1.39 ± 0.03	1.47 ± 0.03	1.27 ± 0.03
42	1.94 ± 0.00	1.39 ± 0.05	1.47 ± 0.03	1.23 ± 0.03
56	1.96 ± 0.03	1.31 ± 0.01	1.19 ± 0.06	1.39 ± 0.03
70	1.94 ± 0.00	1.39 ± 0.03	1.27 ± 0.04	1.39 ± 0.04
84	1.96 ± 0.03	1.31 ± 0.01	1.19 ± 0.03	1.39 ± 0.02

**Figure 2:**
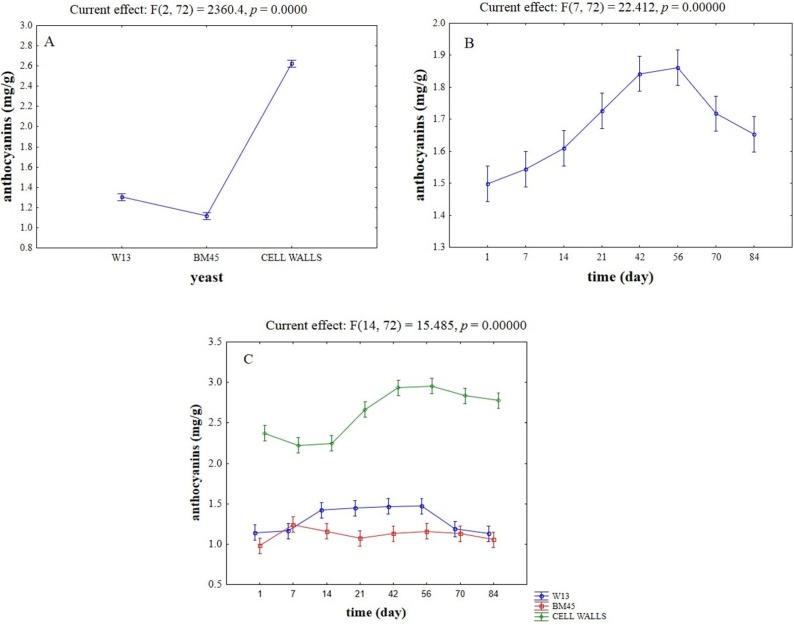
Effective hypothesis decomposition of two-way ANOVA for the effects of yeast/time on the content of anthocyanins on pellets (mg/g). (**A**) Effect of yeasts/yeast cell walls; (**B**) effect of time; and (**C**) interaction.

### 2.2. Evaluation of Potential Competitive Phenomena between OTA, Yeast Cells, and Anthocyanins

**Figure 3:**
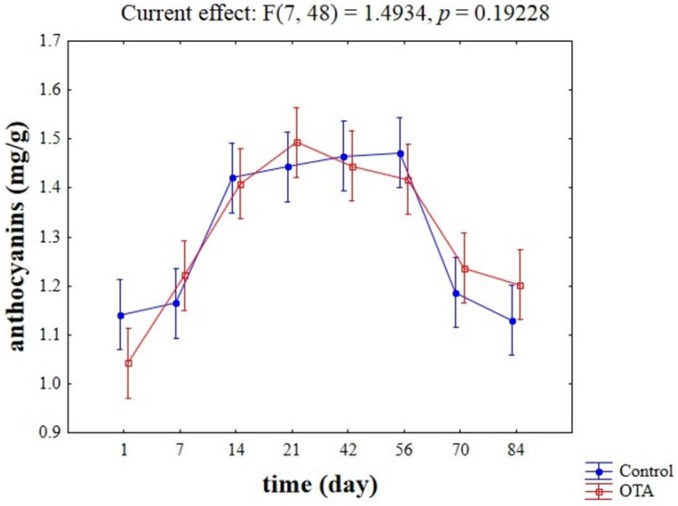
Effective hypothesis decomposition of two-way ANOVA for the effects of absence/presence of OTA on the content of anthocyanins on the pellet of the strain W13 (mg/g).

Figure 2 shows the result of two-way ANOVA analysis for anthocyanins adsorption on yeast cells. Statistical analysis revealed that the kind of adsorbent, time, and their interactive effect were significant (*p* < 0.01). The highest adsorption values were obtained for the yeast cell wall preparation (*ca*. 2.6 mg/g), whereas the lowest adsorption was detected in the case of the commercial isolate BM45. Moreover, the highest level of adsorption (*ca*. 1.8–1.9 mg/g) was found after 42 and 56 days (Figure 2B). Another interesting result was the significant interaction of the adsorbing tool (yeast or yeast cell wall) *vs.* time (Figure 2C), with a strong effect of time on the adsorption ability of yeast cell walls (after 21 days). A significant interactive effect was also found for the strain W13, as the amount of the adsorbed anthocyanins increased after 14 days and then decreased after 70 days. A second statistic revealed that OTA did not affect the adsorption of anthocyanins on yeast cell walls; Figure 3 shows the trend for the strain W13.

### 2.3. Effects of the Different Aging Techniques on Total Anthocyanins Content and Chromatic Characteristics of Wines

**Figure 4:**
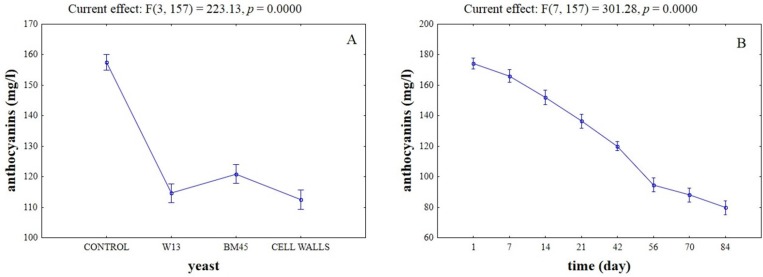
Effective hypothesis decomposition of two-way ANOVA for the effects of yeast/time on the content of anthocyanins in wine (mg/L). (**A**) Effect of yeasts/yeast cell walls; and (**B**) effect of time.

**Figure 5:**
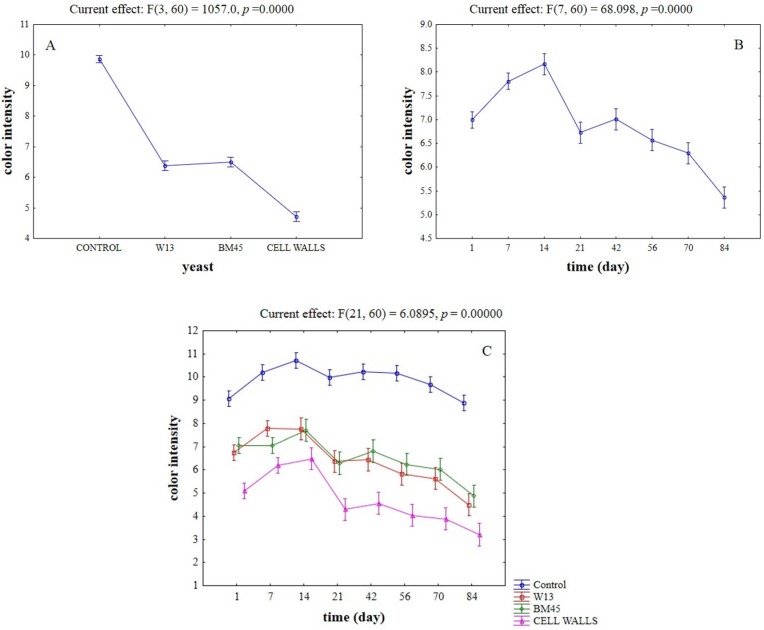
Effective hypothesis decomposition of two-way ANOVA for the effects of yeast/time on color intensity of wines (A_420_ + A_520_ + A_620_). (**A**) Effect of yeasts/yeast cell walls; (**B**) effect of time; and (**C**) interaction.

After assessing OTA fate in wine and how OTA and anthocyanins interacted on cell walls, the second step of this research was aimed at studying the trends of anthocyanins, color intensity, and tonality of wines as a function of OTA and yeasts. First we focused on the impact of adsorbing material (yeasts or yeast cell walls) on anthocyanin content. The actual values of these compounds throughout time are in Table 2. Figure 4 shows the result of two-way ANOVA analysis for the changes in anthocyanins. Control wine was used as reference. Statistical analysis revealed that the kind of adsorbent, and time, affected the content of these compounds (*p* < 0.01). Wines supplemented with yeast cell walls showed a significantly lower content of anthocyanins (effect of adsorbing tool), whereas no significant differences between the different adsorbing tools were found (Figure 4A). As expected, the content of anthocyanins suffered a stronger reduction throughout time (Figure 4B); no interactive effects were found (data not shown). The practical implication of anthocyanins adsorption can be clearly observed in Figure 5, which shows the change in color intensity. Yeast cell walls showed a significant decrease in color intensity, whereas no significant differences between the strain W13 and the commercial isolate BM45 were found (Figure 5A). As expected, the parameter suffered a stronger reduction at the end of the aging (Figure 5B). Another interesting result was the significant interaction of the adsorbing tool *vs.* time, with a strong effect of time on the reduction of color intensity by the yeast cell wall preparation (Figure 5C).

**Table 2:** Concentrations of anthocyanins (mg/L) in control wines and samples supplemented with yeasts or yeast cell walls, with or without OTA (2 μg/L). Mean values ± standard deviation.

**Time (Day)**	**Control**		**W13**	
	**no OTA**	**OTA**	**no OTA**	**OTA**
**1**	191.82 ± 20.03	187.18 ± 25.22	172.41 ± 18.29	167.56 ± 15.54
**7**	186.90 ± 15.31	184.34 ± 3.39	154.52 ± 6.15	160.39 ± 11.12
**14**	174.37 ± 7.28	173.02 ± 8.09	137.24 ± 4.54	144.92 ± 8.12
**21**	171.54 ± 7.57	161.97 ± 8.26	124.31 ± 5.65	127.74 ± 4.85
**42**	152.77 ± 8.36	152.94 ± 6.52	103.18 ± 7.22	102.38 ± 3.71
**46**	136.70 ± 5.93	142.03 ± 3.97	83.07 ± 2.69	84.69 ± 5.83
**70**	128.82 ± 3.94	130.98 ± 2.25	72.26 ± 2.43	77.41 ± 3.74
**84**	117.10 ± 5.47	116.56 ± 1.17	70.14 ± 3.17	71.55 ± 4.37
**Time (Day)**	**BM45**		**Cell Walls**	
	**no OTA**	**OTA**	**no OTA**	**OTA**
**1**	166.75 ± 11.04	166.15 ± 23.48	166.05 ± 18.04	155.33 ± 11.85
**7**	160.57 ± 10.96	163.32 ± 7.84	161.90 ± 17.03	150.79 ± 6.25
**14**	148.36 ± 5.92	143.31 ± 6.81	148.16 ± 9.53	143.71 ± 5.04
**21**	123.90 ± 5.30	134.82 ± 7.93	125.92 ± 3.33	128.55 ± 9.08
**42**	111.93 ± 3.96	112.99 ± 6.24	112.18 ± 9.13	109.30 ± 10.91
**46**	91.97 ± 4.16	88.83 ± 5.59	67.00 ± 3.09	80.14 ± 1.88
**70**	84.49 ± 2.45	86.00 ± 5.42	66.80 ± 5.45	73.47 ± 5.45
**84**	79.13 ± 2.22	79.74 ± 3.24	52.65 ± 2.96	55.79 ± 2.31

**Figure 6:**
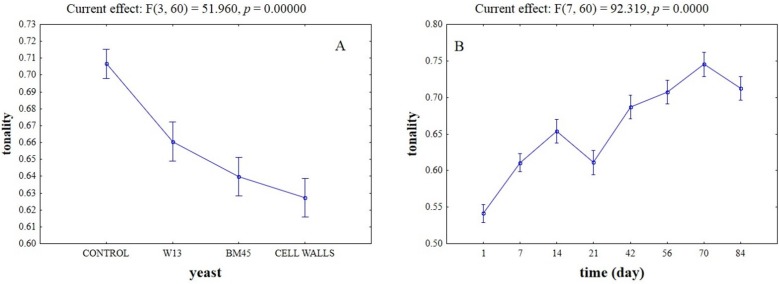
Effective hypothesis decomposition of two-way ANOVA for the effects of yeast/time on tonality of wines (A_420nm_/A_520nm_). (**A**) Effect of yeasts/yeast cell walls; and (**B**) effect of time.

Figure 6 reports two-way ANOVA for the tonality, which decreased stronger in the samples treated with the different adsorbing tools, rather than in the control wine (Figure 6A) and increased throughout time (Figure 6B).

**Figure 7:**
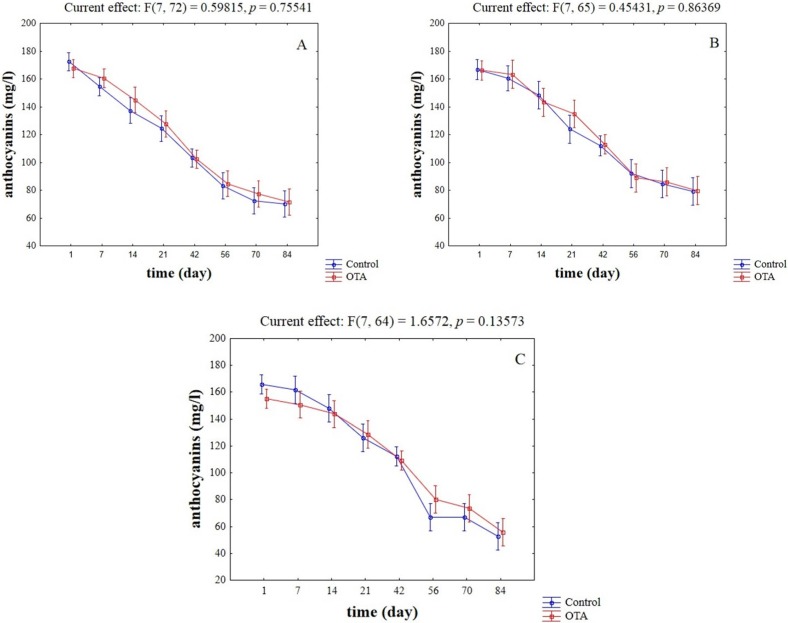
Effective hypothesis decomposition of two-way ANOVA for the effects of absence/presence of OTA on the content of anthocyanins in wine (mg/L). (**A**) Strain W13; (**B**) strain BM45; and (**C**) yeast cell walls.

The last statistic test was conducted to assess if OTA could influence anthocyanin content: OTA was never significant, as showed by hypothesis decomposition for the strain W13 (Figure 7A), BM45 (Figure 7B), and yeast cell wall preparation (Figure 7C).

## 3. Discussion

Although several physical, chemical, and biological methods are available to control the levels of OTA in musts and wines [32], parietal adsorption of toxin by yeasts, mainly *S. cerevisiae*, is considered a promising solution, since it is possible to attain the decontamination without using harmful chemicals and without losses in nutrient value or palatability of decontaminated products [16].

OTA and yeast cell walls could interact due to the chemical traits of both of them. OTA is a complex organic compound, consisting of chlorine-containing dehydroisocoumarin linked through the 7-carboxyl group to 1-β-phenylalanine. Phenol and carboxyl are the main functional groups involved in some different adsorption mechanisms [16]. *S. cerevisiae* cell wall is described in terms of three layers, an outer electron-dense layer, an adjacent less dense layer, and another dense layer bordering the plasma membrane. The yeast wall is composed of a three-dimensional internal skeletal layer of 1,3-β-glucan and 1,6-β-glucan (30%–40% of wall mass) stabilized by hydrogen bonds. Other important components of yeast wall are the mannoproteins (30%–40% of wall mass), which are the most highly-exposed cell-wall molecules, and can form sorption sites. These components are all interconnected by covalent bonds. Mannoproteins are bonded by a 1,6-β-glucan chain with 140 glucose residues to a 1,3-β-glucan chain of approximately 1500 sugar residues [33].

The most recent winemaking technologies are focused on the use of inactivated yeast fractions (e.g., inactivated whole yeasts, yeast autolysates, yeast extracts, cell walls, or mannoproteins) to reduce the time required to obtain wines with physico-chemical and sensory characteristics similar to those aged on lees [2,34]. To address this important topic, two strains of *S. cerevisiae* (the wild strain W13, and the commercial isolate BM45), previously inactivated by heat, and a commercial yeast cell wall preparation have been tested for their OTA-adsorption ability. Yeast cell walls revealed the highest adsorption values in comparison to thermally-inactivated cells (50 *vs*. 43% toxin reduction). Probably, heat treatment caused the inactivation of the metabolism, as well as a possible damage to cell structure. This injury could in turn cause a loss of some cell wall components involved in interactions with OTA and/or to an important alteration of their three-dimensional structure, affecting the accessibility to interaction sites and OTA adsorption within the wall network thickness. Although yeast cell walls removed more OTA than whole yeasts, there is another main trait to keep in mind for the final selection and choice amongst the aging tools, *i.e.*, yeasts or yeast cell walls elimination. Removing the yeast cell wall fraction from wine is more difficult than eliminating whole yeasts, which only require filtration through a filter of 0.45-μm pore size, common in the cellars [7].

Concerning the removing percentages of OTA found in this research, these values were in line with previous reports. For example, Piotrowska *et al.* [35] added a thermally inactivated biomass of *S. cerevisiae* yeast to wines from white grape and blackcurrant juices and decreased after 24 h the content of OTA by *ca*. 60%. Similarly, heat-treated yeast cells have been used by Var *et al.* [36] to remove OTA (a maximum of 30.45% within 4 h) from white wine, but in both studies the time of contact between the yeast and the OTA was shorter than in our research.

An important issue to be addressed is if OTA adsorption could compete with the removal of some wine components, namely anthocyanins.

Although numerous studies have clearly proven the interaction of yeast cells with a variety of compounds, the deep understanding of phenomenon is a challenge since it is driven by a complex interaction solute/solvent, solute/surface, surface/solvent physic-chemical interactions, and solvent cohesion. The affinity for the surface (initial adsorption) as well as the maximum adsorbed amount relies upon the number of binding sites, the accessibility to them, a possible conformational rearrangements of the solute when dealing with polymers and lateral interactions between adsorbed species [2].

The results of this research suggested that OTA and anthocyanins adsorption were not competitive phenomena, as they probably acted in different ways and impacted on different targets on yeast cell wall. At a molecular level, cell wall mannoproteins play a major role in the adsorption of toxin [16], although other parietal components could be involved. For example, some authors reported that a mixture of chitin and β-glucan as well as their hydrolysates removed OTA by 64% to 74% [37]. Similarly, anthocyanins adsorption by yeasts is attributed to cell walls. Morata *et al.* [4] studied the adsorption of anthocyanins by the cell wall of different strains of *Saccharomyces* spp. throughout fermentation, and found that some strains showed removal rate two-fold higher than the others. Unfortunately, information concerning anthocyanin interactions with mannoproteins throughout wine aging remains scarce and is missing for β-glucans and chitins.

A different explanation for the not competitive nature of adsorption phenomenon between OTA and anthocyanins could rely upon the possibility of partial intracellular penetration of these compounds into the whole yeasts [7] and a consequent interaction with the plasma membrane lipids [38]. This hypothesis has been also suggested by Pradelles *et al.* [14] to explain the adsorption of geosmin by autolysed cells. The passage of anthocyanins through the cell wall to the periplasmic space and their interaction with the plasma membrane could also explain the lower adsorption by whole cells than the commercial yeast cell wall preparation.

We could also suggest that the adsorption of anthocyanins on yeast cells in a complex polyphenolic environment does not rely upon a simple adsorption mechanism [6]. Mechanisms of adsorption and complexation (with other biopolymers such as proteins and polysaccharides) could interact, with significant effects by hydrophobic attraction and hydrogen links.

The last issue relies upon the effect of yeast cell wall on the qualitative traits of wine. It is well known that the addition of adsorbing tools such as yeast cells to wine causes important reductions in anthocyanin content, leading to color losses [13]. Thus, the selection of suitable yeasts could also address this trait and avoid the use of strains with high adsorption levels of anthocyanins.

Further investigations are required to elucidate some critical issues, like the effect of the concentration of cell wall material and yeasts; hereby, we focused on a standardized protocol and used the weight as a way to compare the treatments. Another issue that should be addressed is if the preliminary treatment of yeasts or cell walls could affect the adsorption properties towards OTA and anthocyanins.

## 4. Experimental Section

### 4.1. Yeast Strains

The yeasts used in this study were (1) *Saccharomyces cerevisiae* W13 (Accession number: KC542799), a yeast strain belonging to the Culture Collection of the Laboratory of Predictive Microbiology (Department of the Science of Agriculture, food and Environment, University of Foggia, Foggia, Italy) and selected for its OTA-removal ability in a model wine system [28], semi-synthetic [24] and natural grape must [27], and (2) *Saccharomyces cerevisiae* BM45, a commercial mannoprotein-overproducing yeast strain (Lallemand Inc., Montreal, QC, Canada) commonly used in the over-lees aging of red wines [39,40], and previously studied for its OTA-removal ability in a model wine system [28].

### 4.2. Preparation of Inactivated Yeasts

Yeast biomass was obtained by culturing the strains in YPG medium (yeast extract 10 g/L, Oxoid, Milan, Italy; bacteriological peptone 20 g/L, Oxoid; glucose 20 g/L, C. Erba, Milan, Italy; 12 g/L agar technical no. 3, Oxoid) at 30 °C for 24 h. A loop full of yeast culture was then used to inoculate 250 mL Erlenmeyer flasks containing 100 mL of a model synthetic grape juice medium [41]. The flasks were closed with a Müller valve previously filled with sulfuric acid, and incubated at 25 °C. The fermentation rate was monitored daily by weight loss as a result of CO_2_ escaping from the system, until the weight was constant. Completion of fermentation (less than 2 g/L residual sugar) was confirmed by enzymatic determinations (K-FRUGL; Megazyme, Bray, Ireland). Yeast lees were harvested by centrifugation at 4600 g for 15 min at 4 °C (ALC 4239R centrifuge, ALC, Milan, Italy) and washed three times with 0.9% NaCl in order to obtain a yeast biomass with no nutrient impurities [41]. Yeasts were then inactivated by heating at 80 °C for 24 h in an oven [42] to exclude possible changes in wine composition during the experimental aging [14], as well as the involvement of a potential metabolic conversion of toxin by viable cells [43]. The absence of live yeast populations after heating was confirmed by plate count on YPG agar.

### 4.3. Yeast Cell Walls

A commercial yeast cell wall preparation (Biolees; Laffort, Bordeaux, France), previously studied for its ability to remove OTA in a model-wine system [28], was included in the experiment.

### 4.4. Simulation of Wine Aging

In accordance with the literature [31], 20 g/L dry weight of inactivated yeasts (or yeast cell walls) were suspended in 60-mL flasks containing 50 mL of Nero di Troia red wine (*Vitis vinifera* L.; grape harvest, 2013) previously filtered under vacuum (pore size 0.22-μm; Sigma-Aldrich, Milan, Italy) [44] and supplemented with OTA (2 μg/L; Sigma-Aldrich). Two different controls were prepared: (i) wine supplemented with OTA but without yeasts/yeast cell walls and (ii) wine with yeasts/yeast cell walls but without OTA. All dispensed wines were incubated for 84 days [31] in darkness at 18 °C [45] with a weekly stirring (inversion and shaking for 15 s) to simulate enological *batônnage* [46]. Thus, a total of 216 aging experiments were performed (three independent experiments *per* each adsorbing tool, with or without OTA, for nine sampling points).

The following parameters were assessed: (1) OTA concentration, (2) total anthocyanins, and (3) chromatic characteristics.

### 4.5. Analytical Determinations

#### 4.5.1. Extraction of Anthocyanins Adsorbed by the Yeast Cells

The samples were centrifuged at 4600 *g* for 5 min at 4 °C to separate yeast cells (or yeast cell walls) from wine; then, wines and yeasts were separately treated. Yeasts were washed twice using 10 mL of distilled water and then centrifuged at 4600 *g* for 5 min at 4 °C to eliminate any residual wine. The supernatants formed by the washing waters was generally discarded with the exception of those collected at 56 and 84 days, which were submitted to OTA determination in order to assess any losses during the washing operations.

Anthocyanins adsorbed by yeast cells were then extracted by six washes with 5 mL of ethanol:water:hydrogen chloride 37% (70:30:1 *v*/*v*/*v*), mixed with a Vortex for 30 s. Centrifugation at 4600 *g* followed each wash, and the supernatant was kept to obtain 30 mL of yeast extract.

#### 4.5.2. Determination of Total Anthocyanins

Anthocyanins concentration of wines and yeast extracts were spectrophotometrically determined (Cary 50 SCAN UV–Visible spectrophotometer; Varian, Palo Alto, CA, USA) according to the methods of Di Stefano *et al.* [47] and Di Stefano and Cravero [48]. When necessary, wines and/or yeast extracts were opportunely diluted with aliquots of a solution of ethanol:water:hydrogen chloride 37% (70:30:1). Absorbance spectrum between 230 and 700 nm was recorded. The total anthocyanin contents were determined at 540 nm. The results were expressed as mg of malvidin-3-*O*-glucoside chloride per liter of wine (m/L) or per gram of yeast (m/g).

#### 4.5.3. Determination of Chromatic Parameters

Chromatic parameters were determined according to the Glories [49] method. The color intensity was given by the sum of the absorbance to 420, 520, and 620 nm or A_420_, A_520_, and A_620_. Color tonality was expressed by the ratio of the A_420_ and A_520_. Determination of chromatic parameters was done spectrophotometrically using quartz cells of 0.1 cm path length.

#### 4.5.4. Quantification of OTA Concentration

The concentration of OTA in wines, in yeast extracts, and in the distilled-water used to wash the yeast cells was determined using an enzyme linked immuno-sorbent assay (ELISA) method, after immunoaffinity column (IACs) clean-up. A RIDASCREEN^®^Ochratoxin A 30/15 ELISA kit (Art. No. R1311) and RIDA^®^Ochratoxin A column (Art. No. R1303) were used for the analysis. The test procedures were performed following the protocols provided by the manufacturer (R-Biopharm, Darmstadt, Germany). The optical density of the reaction product was determined using ELISA a 96-well plate reader Model No. 680 (Bio-Rad, Hercules, CA, USA) set to 450 nm wavelength. The results were reported as percentages of OTA removed in wine by yeasts or yeast cell walls.

### 4.6. Statistical Analysis

Data were analyzed through two-way analysis of variance (two-way ANOVA) and Tukey’s test as the *post hoc* comparison test (*p* < 0.01), using the software STATISTICA for Windows (StatSoft, Inc., Tulsa, OK, USA; software version 10.0.1011.0).

## 5. Conclusions

The results of this paper suggested that OTA and anthocyanin adsorption were not competitive phenomena, as they probably acted in a different way and impacted on different targets on yeast cell wall. Yeasts removed OTA in an early stage, as significant performances were found just after one day. Longer treatments (>56) hardly increased the removal rates. A challenge was that the addition of yeast cells to wine caused important reductions in anthocyanins content, leading to color loss. This result suggested that the studied yeasts could be proposed as adsorbing tools for short treatments and their use for wine aging could be hardly proposed. Finally, proper selection of yeast for wine aging, with the functional trait of toxins removal, should take into account parietal adsorption of phenols in order to minimize their impact on wine attributes.
